# Effects of Different Dietary Vegetable Lipid Sources on Health Status in Nile Tilapia (*Oreochromis niloticus*): Haematological Indices, Immune Response Parameters and Plasma Proteome

**DOI:** 10.3390/ani10081377

**Published:** 2020-08-08

**Authors:** Chatsirin Nakharuthai, Pedro M. Rodrigues, Denise Schrama, Suksan Kumkhong, Surintorn Boonanuntanasarn

**Affiliations:** 1 School of Animal Technology and Innovation, Institute of Agricultural Technology, Suranaree University of Technology, 111 University Avenue, Muang, Nakhon Ratchasima 30000, Thailand; chatsirin_nak@sut.ac.th (C.N.); M5730049@g.sut.ac.th (S.K.); 2 Universidade do Algarve, Centro de Ciências do Mar do Algarve (CCMAR), Campus de Gambelas, Edificio 7, 8005-139 Faro, Portugal; pmrodrig@ualg.pt (P.M.R.); dschrama@ualg.pt (D.S.)

**Keywords:** proteomics, immune, haematology, plasma, saturated fatty acids, n6-PUFAs, n3-PUFAs, Nile tilapia, gene expression

## Abstract

**Simple Summary:**

This study presents the effects of different dietary vegetable lipid sources (DLs) on several blood parameters, which may indicate the health status of Nile tilapia (*Oreochromis niloticus*). The tested DLs had no effects on haematological indices. However, the immune response analysis showed that DLs promoted immune systems via different processes. Dietary polyunsaturated fatty acids (PUFAs) increased expression of several proteins involved in coagulation activity, while saturated fatty acids (SFAs) might influence heme lipid-oxidation. Overall, different DLs had effects on several parameters corresponding to health status in Nile tilapia, and dietary PUFAs appear to promote health in this fish.

**Abstract:**

This study aimed to investigate the effects of DLs, including palm oil (PO; an SFAs), linseed oil (LO; n-3 PUFAs) and soybean oil (SBO; n-6 PUFAs) on the health status of Nile tilapia (*Oreochromis niloticus*) during adulthood. Three experimental diets incorporating PO, LO or SBO were fed to adult Nile tilapia for a period of 90 days, and haematological and innate immune parameters were evaluated. Proteome analysis was also conducted to evaluate the effects of DLs on plasma proteins. The tested DLs had no significant effects on red blood cell (RBC) count, haematocrit, haemoglobin, and total immunoglobulin and lysozyme activity. Dietary LO led to increased alternative complement 50 activity (ACH50), and proteome analysis revealed that PO and SBO enhanced A2ML, suggesting that different DLs promote immune system via different processes. Dietary LO or SBO increased the expression of several proteins involved in coagulation activity such as KNG1, HRG and FGG. Increased HPX in fish fed with PO suggests that SFAs are utilised in heme lipid-oxidation. Overall, DLs with distinct fatty acids (FAs) affect several parameters corresponding to health status in Nile tilapia, and dietary LO and SBO seemed to strengthen health in this species.

## 1. Introduction

Dietary lipids (DLs) are important sources of metabolic energy, which provide essential fatty acids (FAs) and assist in carrying and absorbing fat-soluble vitamins. Adequate FAs is one of the most important factors influencing various biological processes, such as growth, reproduction and immune responses [1,2]. Furthermore, polar lipids and particularly phospholipids are the main components of the cellular membrane and are responsible for maintaining its flexibility and structure. Eicosanoids are molecules derived from 20-carbon (n-3 and n-6) Polyunsaturated fatty acids PUFAs. Eicosanoids consist of prostanoids, prostacyclins, thromboxanes and leukotrienes, which act as bioactive signalling lipids to regulate inflammation and immunity [1,3,4]. DLs have been shown to affect haematological parameters, such as red blood cell (RBC) number, the haematocrit and haemoglobin, which are essential in vertebrates, particularly in fish [5,6]. Lipids are involved in heme-driven oxidative stress [7]. The effects of DLs in both FA composition and the level of intake is related to blood coagulation [8]. DLs or FAs are; therefore, crucial for the correct functioning of a number of vital physiological processes, which significantly influence the health of vertebrates.

In inland aquaculture, tilapia is the second most important finfish species cultured worldwide, with Nile tilapia (*Oreochromis niloticus*) dominating global tilapia production. The global production of tilapia was reported to be 5.89 metric tonnes (mt) in 2016 [9] and is estimated to constitute 62% of total global aquaculture production in 2030 [10]. Nile tilapia is an omnivorous fish, which use carbohydrates as their primary energy source. However, DL are necessary to provide essential fatty acids (EFAs) for various physiological processes, in order to achieve normal growth and maintain their health status. For example, in Thailand, commercial diets for Nile tilapia, from fingerlings to marketable size, contain 4%–5% of DL, 31%–41% of carbohydrates and 25%–40% of protein. The recommended levels of DL are between 5% and 12%, and EFAs, including linolenic acid (C18:3n-3) and linoleic acid (C18:2n-6), are required for tilapia. In addition, the requirement for dietary n-6 PUFAs has been estimated at 0.5% to 1% [11]. Like other freshwater fish, Nile tilapia is able to synthesize n-6 LC-PUFAs and n-3 LC-PUFAs [12,13]. Therefore, incorporation of proper vegetable oil in diets for Nile tilapia is adequate without n-3 LC-PUFA supplementation, such as eicosapentaenoic acid (EPA) and docosahexaenoic acid (DHA). Extensively, most feed fats used from vegetable oils in aquaculture include palm oil (PO), soybean oil (SBO) and linseed oil (LO). Previous studies of Nile tilapia during adulthood have demonstrated that different DLs including saturated fatty acids (SFAs) in PO, n-6 PUFAs in SBO and n-3 PUFAs in LO result in normal growth with no sign of abnormalities. Nevertheless, more studies of health impact of DLs are required for further applications for benefits associated with DLs in Nile tilapia.

Recently, several molecular biology techniques have been applied to gain insight into the health impact of DLs. For example, the gene encoding delta-6 desaturase (*fads*2) was cloned in Nile tilapia, and was demonstrated to be able to convert linoleic acid (C18:2n-6) and α-linolenic acid (C18:3n-3) to gamma-linolenic acid (C18:3n-6) and stearidonic acid (C18:4n-3), respectively [14]. Evaluation of the expression of cyclooxygenase (*cox*-*2*) (overexpression) and eicosanoid receptor (*ep4*) (down-regulation) genes was used to reveal the inductive effect of SBO-based diet on inflammatory-like process in gut of Senegalese sole (*Solea senegalensis*) [15] In addition, epigenetic analysis was conducted to interpret that methylation in the promoter of *fads*2 in offspring was correlated with DL of broodstock [16]. In this study, a high-throughput proteomics technology was used to enable a comprehensive investigation into the biological processes affected by dietary intake of FAs. Proteomics is a useful tool in nutritional research as it can provide large-scale information on the effects of particular nutrients on physiologic and metabolic pathways [17,18,19]. Previous proteomic analysis of liver tissue revealed that different DLs, including SFAs, n-6 PUFAs and n-3 PUFAs, resulted in distinct protein expression in liver tissue. While dietary SFAs provide an energy source, dietary n-6 PUFAs and n-3 PUFAs appeared to be involved in sustaining normal health [18].

To determine how DLs influence the health status of Nile tilapia, we investigated the effects of palm oil (PO), linseed oil (LO) and soybean oil (SBO) on haematological and immune parameters. Growth also was determined. In addition, a comparative proteomic analysis of plasma was carried out to evaluate the effect of DLs on the overall health. Moreover, the effects of DL sources on mRNA levels corresponding to proteins identified as being significantly different in the proteome analysis were determined.

## 2. Materials and Methods

### 2.1. Experimental Design, Diet Formulation and Fish Culture

All experimental protocols were accepted by the Ethics Committee of Suranaree University of Technology, Animal Care and Use Committee (approval No. 1731050). In addition, organisms handling and subsequent procedures complied with European laws (2010/63/EU) and Portuguese legislation for the use of laboratory animals (DL n°113/2013, 7 August). Male tilapia (n = 120) (initial body weight 221.7–275.8 g) were maintained at Suranaree University of Technology (Nakhon Ratchasima, Thailand). Ten fish were randomly distributed into each of the 12 cement ponds (1.8 × 1.8 × 0.8 m^3^). Fish were acclimatised to the experimental conditions for two weeks and cultured under continuous aeration, and a flow-through water change system, implemented to replace one-third of the water in each pond with de-chlorinated water every week.

To test the effects of feed fats used in aquaculture, three different enriched DLs (PO, LO or SBO) diets were fed to fish, randomly allocated to the experimental ponds (four replicates per DLs treatment, n = 40). Table 1 shows the composition of the experimental diets. All feed ingredients were obtained from commercial companies and mixed to produce pelleted diets. The composition of moisture, crude protein, crude fat and ash content were determined following the standard methods [20]. The experimental feeds were stored at −20 °C until use. Fish were fed the experimental diet until satiety twice daily for 90 days. By the end of this period, weight gain, feed intake and survival rate were measured. Throughout the experimental period, ponds were kept at natural temperature (27–28 °C) and photoperiod, dissolved oxygen at 4.68–6.84 mg L^−^^1^ and pH at 7.19–8.54.

### 2.2. Fish Sampling and Blood Collection

At the end of the experimental period (90 days), fish were starved for 17 h prior to sampling of blood, liver and muscle samples. Two fish per tank (n = 8 per treatment) were used for haematological indices and innate humoral parameters. Fish were euthanized with 2-phenoxyethanol (0.2%) and blood samples were collected from the caudal vein of fish using a 21-gauge needle and transferred into a tube containing 1% of 15%EDTA (*v*/*v*) for determination of haematological indices. Blood plasma for determination of humoral innate immune parameters was collected by centrifugation at 9000× *g* for 10 min at 4 °C. In addition, one fish from each tank (n = 4 per treatment) was collected for proteomic analysis and gene expression determination. Fish were euthanized, and blood was collected into a tube containing 1% of 15% EDTA (*v*/*v*). After centrifugation at 9000× *g* for 10 min at 4 °C, blood plasma was collected for proteome analysis. After bleeding, the liver and muscle were collected for qRT-PCR analysis. All samples were frozen first in liquid nitrogen and then kept at −80 °C until further use.

### 2.3. Haematological and Immune Assays

Immediately following the blood sampling, EDTA blood was used for analysis of haematological parameters. RBC count, haematocrit and haemoglobin were measured, following methods described by Tiengtam et al. [21]. Immune parameters, including total immunoglobulin, lysozyme activity and alternative complement haemolytic 50 (ACH50) activity, were also measured. Total immunoglobulin (Ig) was determined using a modified method previously described [22,23]; the total protein (Biuret method; Erba, Mannheim) was used for total Ig measurement. Briefly, 10 µl of diluted plasma was mixed with an equal volume of 12% PEG solution (polyethylene glycol, Sigma) in a 1.5 mL microcentrifuge tube. Incubation was performed at room temperature (25 °C) for 30 min and centrifuged at 3000× *g* for 5 min at 4 °C, to precipitate the immunoglobulin. Next, 4 µl of supernatant (non-Igs), diluted plasma (total protein) and standard bovine serum albumin (Sigma) were placed into a flat-bottomed 96-well plate in triplicate and protein content was determined following the manufacturer’s instructions. The optical density (OD) was read at 546 nm in a plate reader (BioTek^TM^ EPOCH). Total Igs were calculated by subtraction of the total protein from non-Igs after precipitation. Protein concentrations were obtained from the standard curve made with bovine serum albumin.

Serum lysozyme activity was determined using a turbiditic assay as previously described [22,24] with some modifications. Briefly, different concentrations of lyophilized hen egg white lysozyme (0, 2.5, 5, 10, 15 and 20 µg mL^−1^) in 0.06 M phosphate citrate buffer, pH 6.0 were used as reference standards to construct a standard curve. Nile tilapia plasma (10 µL) and each concentration of standard lysozyme were added into wells of a flat-bottomed 96-well plate, in triplicate. To initiate enzymatic assay, a suspension of 190 µl of 0.2 mg/mL dried *Micrococcus lysodeikticus* (Sigma-Aldrich, Germany) cells was added into all the wells to a final volume of 200 µl per well. The reaction was carried out at room temperature (25 °C) and OD read at 450 nm at times 0 and 30 min. Nile tilapia lysozyme concentrations were quantified from a standard curve of known hen egg white concentrations.

ACH50 activity ACH50 was measured using the method of Milla et al. [25] with modifications. Briefly, goat red blood cells (GRBC) were used as targets at a final concentration of 5 × 10^7^ cells/mL. Triplicates of tested plasma (50 µL) as a complement source were 2-fold serial diluted in EGTA-GVB buffer (gelatin veronal buffered saline, 10 mM ethyleneglycol-bis (beta-amino-ethyl ether) N-N’-tetraacetate). Then, 50 μL of GRBC suspension was added to the diluted plasma and incubated for 90 min at room temperature (25 °C). Mixtures were then centrifuged at 3000× *g* for 10 min at 4 °C. The OD of the supernatant was read at 415 nm. The volume of complement causing 50% haemolysis (ACH50) of GRBCs was determined, and the number of ACH50 units/mL was calculated for each experimental group.

### 2.4. Plasma Proteome Analysis

#### 2.4.1. Protein Preparation, Labelling and Two-Dimension Gel Electrophoresis

Protein quantifications were carried out with Quick Star^TM^. Bradford Protein Assay using bovine serum albumin (Bio-Rad Laboratories) as a standard. Plasma samples were diluted for concentration determination in DIGE lysis/labelling buffer containing 7 M urea, 2 M thiourea, 30 mM Tris0HCl (pH 8.5), 1 mM EDTA, 4% *w*/*v* CHAPS (3-((3-cholamidopropyl) dimethylammonio)-1-propanesulfonate), and 1% *v*/*v* protease inhibitor cocktail (Sigma-Aldrich Corporation, St. Louis, MO, USA). To perform DIGE minimal labelling, 50 µg of proteins (pH adjusted to 8.5 by addition of 0.3 M NaOH) of each sample were labelled with 400 pmol of fluorescent amine reactive cyanine dyes reconstituted in anhydrous dimethyl formamide, following the manufacturer’s instructions (5 nmol labelling kit, GE Healthcare, Little Chalfont, UK). Labelling was carried out according to a previously described method [18]. Samples were randomly labelled with Cy3 and Cy5 to prevent confounding of an eventual “dye effect” with the biological effect. As an internal control, equal quantities of protein from all samples were pooled and labelled with Cy2. Subsequently, a mixture of labelled proteins (50 µg) of one sample from each dietary treatment and 50 µg of internal standard were added in rehydration buffer (8 M urea, 2% *w*/*v* CHAPS, biolyte (pH 3-10) and 0.2% (*w/v*) dithiothreitol) to a final volume of 450 µL and then applied to 24 cm Immobiline^TM^. Drystrip gels, pH 4-7 linear gradient (GE Healthcare). Subsequently, passive rehydration was performed for 15 h on 24 cm Immobiline^TM^ Drystrips (GE Healthcare, Little Chalfont, UK) with linear pH 4-7. Isoelectric focusing (IEF) was conducted in 5 steps: 500 V gradient 1 h, 500 V step-n-hold 1 h, 1000 V gradient 1 h, 8000 V gradient 3 h and 8000 V step-n-hold 5 h for a total of 60,265 Vhr using Ettan IPGphor at 20 °C (GE Healthcare, Little Chalfont, UK). Focused strips were reduced with 6 mL of equilibration buffer (50 mM Tris-HCl pH 8.8, 6 M urea, 30% (*v*/*v*) glycerol and 2% SDS) with 1% (*w*/*v*) dithiothreitol for 15 min in dark. Subsequently, the focused strips were alkylated with 6 mL of equilibration buffer with iodoacetamide (IAA) for 15 min in dark. For the second dimension, the strips were then loaded onto 12.5% Tris-HCl SDS-PAGE gels and ran in an Ettan DALTsix Large Vertical System (GE Healthcare, Little Chalfont, UK) at 10 mA/gel for 1 h followed by 60 mA/gel until the bromophenol blue line reaches the end of the gel in dark, using a standard Tris-Glycine-SDS running buffer.

#### 2.4.2. Gel Image Acquisition and Analysis and Protein Identification by MALDI-TOF/TOF

Following electrophoresis, all gels were scanned with a Typhoon Trio^TM^ Variable Mode Imager (GE Healthcare) using three laser emission filters (520BP40 for Cy2, 580BP30 for Cy3 and 670BP30 for Cy5) with a photomultiplier voltage of 540V, at a resolution of 100 µm. Image analysis was conducted using the SameSpots^TM^ software (Totallab, Newcastle-Upon-Tyne, UK). Significant different spots were detected using a one-way ANOVA followed by post-Hoc Tukey and exported as normalized volumes.

Protein spots showing a significant difference in expression between conditions were manually excised from Coomassie blue stained gels and identified at the GIGA proteomics facility (Liege, Belgium). Trypsin in gel digestion and purification of protein samples were performed according to [18]. Protein identification and tandem mass spectrometry (MSMS) analysis was performed on a MALDI-TOF-TOF-MS UltrafleXtreme (Bruker). Automatic spectra acquisition was piloted with the software Flex control^TM^ v 3.4 and real time analysis by Flex analysis^TM^ v 3.4 (Bruker). Database searches were carried out in real time with BioTools^TM^ v 3.2 (Bruker) on the Mascot server v 2.2.06 (Matrix Science, Tokyo, Japan). A SwissProt database search was restricted to Actinopterygii taxonomies, with 100 ppm of mass error tolerance in MS and MSMS precursor and 0.3 Da tolerance on MSMS fragments. A second search was made with the same parameters on National Center for Biotechnology Information (NCBI) database, restricted to Actinopterygii taxonomies.

### 2.5. Quantitative Reverse Transcription Polymerase Chain Reaction (qRT-PCR) Analysis

#### 2.5.1. Molecular Cloning of Genes

The expression of mRNA from the corresponding proteins was analysed using real-time quantitative reverse transcription polymerase chain reaction (qRT-PCR), to investigate whether the effects of DLs are also detectable at the transcript level. Total RNA was extracted from 100 mg of liver using Trizol® reagent (Invitrogen Corporation, Carlsbad, CA, USA) and RNase free DNase I (Promega Corporation, Madison, WI, USA) according to the manufacturer’s instructions. First strand cDNA was synthesized using the ImProm-II^TM^ Reverse Transcription System kit (Promega Corporation). Table 2 shows the primers and the expected sizes of amplicons that were used for amplification of the partial cDNA of target genes. Reverse transcription polymerase chain reaction (RT-PCR) was performed in a total volume of 50 µl, which contained 200 μM of each dNTP, 5 pmol of each primer, 2.5 mM MgCl_2_, 1.0 × buffer Ex Taq^TM^, and 1.25 U Ex Taq^TM^ (Takara Shuzo Co., Ltd., Shiga, Japan). For the PCR reaction, initial denaturation was conducted at 95 °C for 3 min followed by 40 reaction cycles, each consisting of a denaturation step at 95 °C for 30 s, annealing at 55–68 °C for 30 s, and extension at 72 °C for 45 s, with a final elongation step at 72 °C for 10 min. PCR products of the expected sizes were isolated and purified using the UltraClean^TM^ 15 DNA Purification Kit (MO Bio Laboratories, Solana Beach, CA, USA). The PCR-amplified DNA fragments were cloned into the pGEM® T-Easy plasmid (Promega Corporation). The plasmids were sequenced by Macrogen, Inc. (Seoul, Korea) and stored for further use as a standard for quantitative RT-PCR (qRT-PCR).

#### 2.5.2. qRT-PCR Analysis Gene Expression

Total RNA was isolated from 100 mg of liver or muscle (four replicates per treatment), and cDNA was prepared as described above. qRT-PCR amplification (in duplicate) was carried out using LightCycler® 480 SYBR Green I Master Mix (Roche Applied Science, Indianapolis, IN, USA). The *18s rRNA* mRNA was used as an internal reference for normalisation of data. The primers and annealing temperatures used in this study are shown in Table 2. In order to evaluate mRNA levels corresponding to proteins identified as being significantly different in the proteome analysis, the mRNA level of *a2ml, kng1*, *hpx*, *fgg*, *hrg*, *cdh*, *c19orf53* and *znf501* were quantitatively analysed as described in [18].

### 2.6. Statistical Analysis

All statistical analyses (growth rates, haematological and innate immune parameter and mRNA levels) were carried out using SPSS, v 12 (SPSS Inc., Chicago, IL, USA). A one-way analysis of variance was used followed by the post hoc Tuckey’s test on significant values, to rank the groups. Throughout the experiment, effects and differences were considered significant at *p* < 0.05. In addition, mean protein abundance was considered to be significantly different between dietary groups when *q*-value <0.05. Preliminary assessment of data quality was performed using a principal component analysis with auto scaling. Pearson’s correlation coefficient was performed to determine the relationship of the expression of the plasma protein abundance and its corresponding mRNA level in the liver or muscle.

## 3. Results

**Figure 1:**
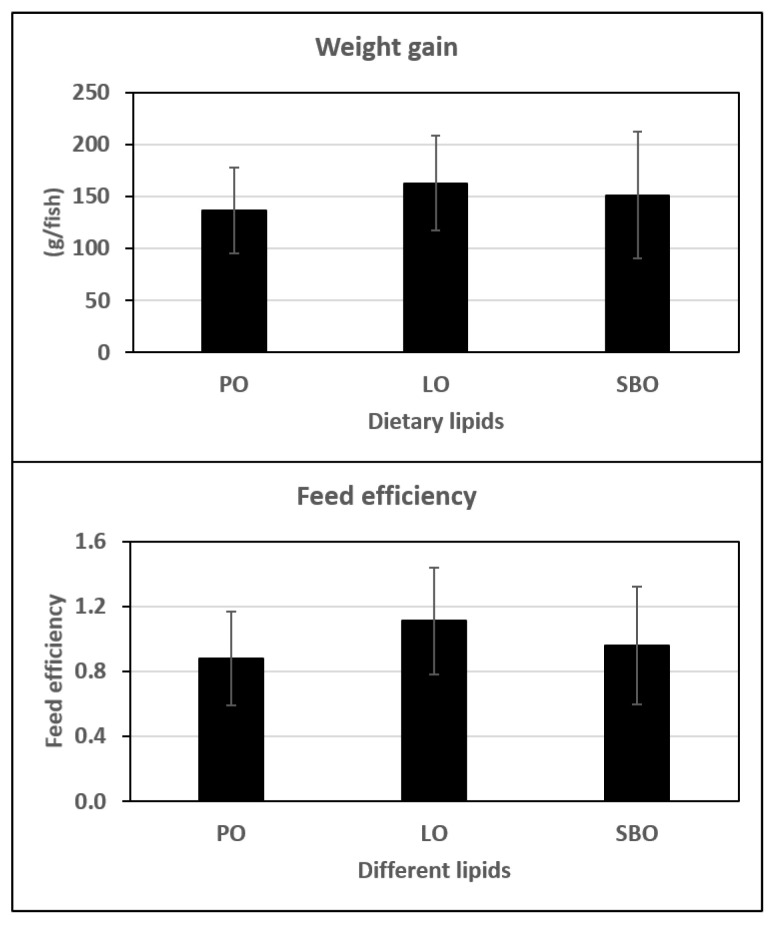
Growth performance including weight gain, specific growth rate and feed efficiency of Nile tilapia fed experimental diets containing palm oil (PO), linseed oil (LO) or soybean oil (SBO) for 90 days. Note that there were no significant differences in growth parameters among experimental diets (*p* > 0.05). Weight gain = final body weight – initial body weight. Feed efficiency = wet weight gain/dry feed fed.

After the experimental period, weight gain and feed efficiency were not different (*p* > 0.05) between the experimental groups (Figure 1). The survival rate of all experimental groups was 100%. The effects of DLs on haematological indices and innate immune parameters are shown in Table 3. There were not differences in RBC counts, haemoglobin and haematocrit among experimental groups (*p* > 0.05). DLs did not significantly influence lysozyme activity or total immunoglobulin (*p* > 0.05). Fish fed dietary LO had significantly higher ACH50 levels (*p* < 0.05) (Table 3).

**Figure 2:**
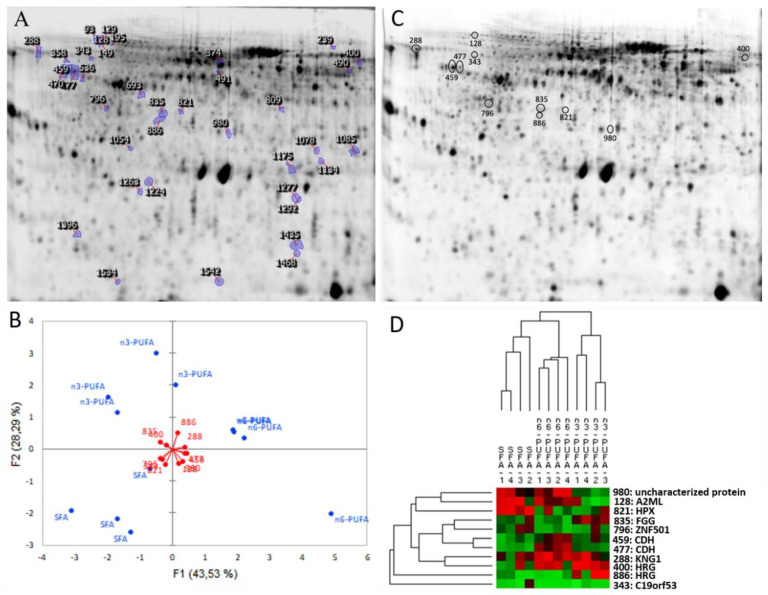
Different abundance of plasma protein identified by 2D-DIGE analysis in Nile tilapia fed experimental diets for 90 days. (**A**) Plasma proteins were separated in pH 4–7 IPG strips for the first dimension and 12.5% polyacrylamide gel electrophoresis for the second dimension. An internal standard in all gels comprised of an equal concentration of proteins from all samples which was labelled with Cy2. Protein spots that show significant differences in abundance between dietary lipids (DLs) are indicated (*p* < 0.05); (**B**) principal component analysis of plasma proteins for each of 12 fish fed DLs including dietary PO (source of SFAs), LO (source of n3-PUFAs) or SBO (source of n6-PUFAs); (**C**) differential abundance of identified protein spots as a result of DLs; (**D**) heat map construction of a hierarchical cluster of 11 proteins that were significant differently according to feeding with different DLs (see Table 4 for protein names of each labelled protein spot).

**Figure 3:**
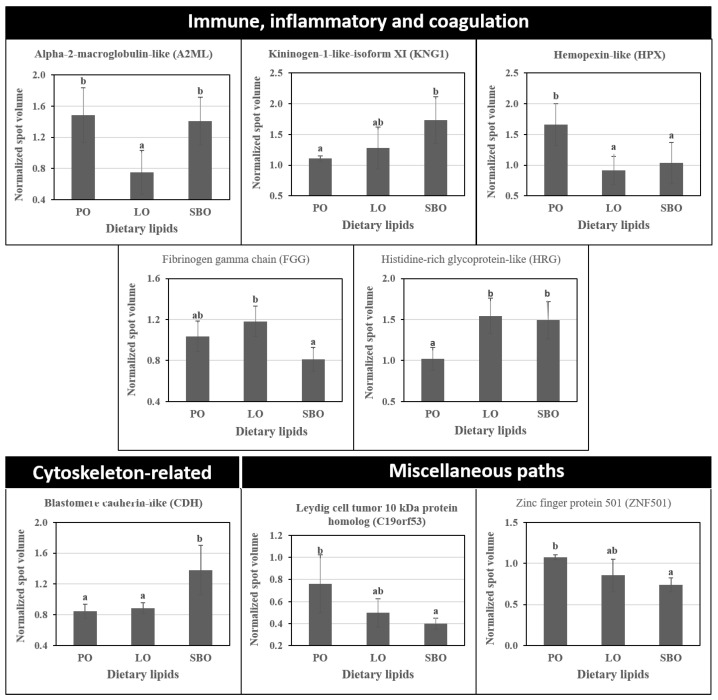
Differential abundance of proteins identified in the plasma of Nile tilapia fed experimental diets containing palm oil (PO), linseed oil (LO) or soybean oil (SBO) for 90 days. Proteins were identified and categorized according to their biological functions: immune, inflammatory and coagulation response; cytoskeleton-related; and miscellaneous biochemical pathways. Note that similar differential abundance of two spots for CDH and HRG were observed. Black bars show the mean normalized value of each protein. Different letters denote significant differences in the abundance of each protein spot between experimental fish fed dietary lipids (*p* < 0.05).

The proteomic analysis of plasma showed that a total of 1307 protein spots were detectable (pH gel range of 4–7). Thirty-nine protein spots were significantly different in their abundances between the different DLs (Figure 2A). PCA analysis of the significantly different proteins shows that three distinct clusters of plasma proteins were associated with different DLs (Figure 2B). Fourteen protein spots were excised from the gels and submitted for protein identification using MS. Subsequently, the Mascot search program was used to compare the mass of the peptide fragment against those in the Genbank database (https://www.ncbi.nlm.nih.gov/genbank/). Protein homologies from protein databases were identified for 4 spots (MS) and 7 (MSMS) spots that were modulated according to the PO, LO, and SBO diets (Figure 2C; Table 4; Table S1). Hierarchical clustering of proteins shows that the relative abundances of proteins were significantly different (*p* < 0.05) depending on the DL source (Figure 2D). Most of the identified proteins were involved in several biological processes including humoral innate immunity, coagulation, and transcription factors. Figure 3 shows the mean normalised values of the identified proteins, including alpha-2-macroglobulin-like (A2ML), kininogen-1-like isoform X1 (KNG1), Leydig cell tumour 10 kDa protein (C19orf53), histidine-rich glycoprotein-like (HRG), blastomere cadherin-like (CDH), zinc finger protein 501-like (ZNF501), fibrinogen gamma chain (FGG) and hemopexin-like (HPX), which were significantly different as a result of PO, LO, and SBO diets (*p* < 0.05). Note that, protein spots (spot no.459 and 477) and (spot No.400 and 886) were identified as CDH and HRG, respectively. In addition, uncharacterized protein was observed for protein spot 980.

In this study, qRT-PCR was carried out to determine the expression levels of transcripts in the liver and muscle, which had been identified from proteomics as being significant modulated by DLs (Table 5). There were significant correlations between hepatic mRNAs and plasma protein levels of *c9orf53* (0.592; *p* *=* 0.043) and *znf501* (0.579; *p* *=* 0.048). Our results showed that increased expression of hepatic *kng1*, *hrg*, *cdh* and *znf501* were observed in fish fed PO. Elevation of muscular *kng1*, *hpx* and *znf501* expression was founded in fish fed SBO or LO (*p* < 0.05). However, DLs had no effect on mRNA levels of *a2ml*, *fgg* and *c19orf53* in either liver or muscle tissue (*p* > 0.05).

## 4. Discussion

DLs are important, not only for providing energy, but also for a number of biological processes. In poikilothermic animals, particularly fish, the nutritional requirement for FAs varies across species. Therefore, DLs, which contain different FAs, influence growth as well as have an impact on an organism’s health. DLs containing PO, SBO and LO contain different FA content, which has an effect on the liver proteome of fish fed the different DLs. Liver function is crucial for a range of vital physiological and metabolic functions [18]. In accordance with other studies, our results showed that DLs had no significant effects on growth, suggesting that the FA content in all experimental diets were adequate for the requirement of EFA for growth in Nile tilapia. In addition, this study examined the effects of DLs by demonstrating how different DL influenced haematological indices and humoral innate parameters. Furthermore, using a proteomic analysis of plasma, we provide evidence of how DL contributes to the health status of Nile tilapia. Although the functions of all the proteins detected are not fully determined, different DLs modulated several proteins that are associated with fish health including coagulation, immunity and oxidative stress. Our findings provide valuable information on the health impacts of DLs containing different FAs.

Haematological parameters are useful indicators of fish health and nutritional status. Several reports demonstrated that DLs had direct effects on membrane lipid composition of RBC which influenced RBC functions [26,27,28]. Our study showed that there were no significant differences in haematological parameters among experimental DL groups. These findings suggested that different DLs containing different composition of FAs had no detectable effects on haematological function in Nile tilapia during adult phase. Similarly, Nile tilapia fed different lipid sources such as LO, corn coil and beef tallow for 12 weeks showed no change in RBC numbers, haematocrit and haemoglobin levels [5]. In addition, Nile tilapia fed with different levels of DL (6%, 10% and 14%); a mixture of corn oil and menhaden fish oil, for 12 weeks showed no variation in these haematological parameters [6]. However, in Nile tilapia, dietary SBO for 60 days led to elevated haematocrit, when compared with fish fed dietary LO, corn oil, fish oil and olive oil [29]. Therefore, in Nile tilapia, different DLs (SFAs, n-6 PUFAs, n-3 PUFAs) have little effect on haematological parameters, and the extent of effect might depend on the duration of feeding.

The innate immune system is a fundamental defence system in fish, particularly humoral innate components [30]. In this study, we evaluated the effects of different DLs on several humoral immune-related parameters such as lysozymes, total immunoglobulin and plasma ACH50. Lysozymes are bacteriolytic enzymes with specific hydrolytic activity, which act against peptidoglycan of bacterial cell walls. Total immunoglobulin, which is a total protein related to immunoglobulin, has been an indicator of immune status in fish. ACH50 acts as a link between the innate and adaptive immune responses, promoting the ability of both antibodies and phagocytic cells to eliminate pathogenic bacteria, damaged cells and foreign cells [reviewed in 30]. Our results showed that although different DLs influenced neither lysozyme nor total immunoglobulin levels, adult fish fed LO had highest ACH50 compared to those fed the others. The positive effects of LO on innate immune response might be due to beneficial influence of n-3 PUFAs on cellular membrane of several immune cells [31], which consequently promote the secretion of humoral innate components. Indeed, DLs containing different FAs showed variable effects on the activities of these innate immune parameters. For instance, there were no significant differences in lysozyme levels in Nile tilapia fed different levels of fish oil and PO, but serum protein was significantly higher with increasing PO levels [32]. Different DLs (6%, 10% and 14%); a mixture of corn oil and menhaden fish oil had no significant effects on lysozyme activity in Nile tilapia. However, increased levels of lipid intake led to an increase in serum protein and decreased alternative complement activity [6]. Reductions in serum protein, lysozyme and natural haemolytic complement activities were measured in Nile tilapia fed beef tallow oil, which was deficient in both n-6 and n-3 PUFAs. Fish fed with different oils containing n-6 and n-3 PUFAs maintained higher levels of these immune parameters [5]. Different DLs (SBO, LO, corn oil, fish oil, olive oil) had no effects on alternative complement activity; however, dietary olive oil led to decreased lysozyme activity in *Nile tilapia* [29]. However, while dietary vegetable oil did not significant influence lysozyme activity, it significantly decreased alternative complement activity in Japanese seabass [33]. Overall, although DLs containing different FAs have variable effects on immune-related responses, depending on the fish species (most likely due to differences in feeding habits), both n-3 and n-6 PUFAs appear to promote these humoral innate immunity parameters.

Studying the proteomic profile in plasma is a valuable tool to investigate the effects of different DLs on the protein-related humoral immune system. A2ML is a major protease inhibitor in plasma and is involved in multiple physiological functions. One of its most important functions is related to humoral innate immunity [34], reviewed in [35]. Indeed, A2ML acts as the cytokine-binding protein in plasma [36]. Serum A2ML upregulation is involved in a metabolic disorder, which could potentially be used as a clinical marker in subjects with diabetes [37]. A proteome analysis comparing fish oil to dietary LO showed a higher expression level of A2ML in the Chinese mitten crab (*Eriocheir sinensis*) after feeding with LO [38]. In contrast, the proteomic analysis presented here shows that dietary PO and SBO increase plasma A2ML levels compared to LO. Despite these contradictory results, proteome analysis has revealed that DLs influence A2ML, and the effects are variable between organisms. In Nile tilapia, dietary n-6 PUFAs and SFAs led to increased plasma levels of A2ML, which would affect humoral innate immunity.

By studying proteomics in plasma, we were also able to investigate the effects of different DLs on other physiological processes, which also influence health status. Our findings demonstrated that different DLs modulated several plasma proteins involved in coagulation and heme-lipid oxidation. For instance, three proteins, which were significantly affected by DLs, were demonstrated as key factors associated with coagulation activity; FGG, KNG1 and HRG. FGG is a gamma component of fibrinogen. Mutations of this protein can lead to hypofibrinogenemia [39]. Kininogen is essential for blood coagulation. Previous investigations demonstrated that high fat feeding decreased kininogen content in the liver of mice, and different FAs differentially regulate expression of kininogen [40,41]. Histidine-rich glycoprotein (HRG) is capable of binding to a wide range of ligands and are able to modulate various biological processes such as blood coagulation, fibrinolysis, antimicrobial activity and innate immune systems [42,43]. This study found that dietary LO and PO led to an increase in FGG in plasma. In addition, fish fed dietary SBO and LO had higher kininogen and HRG compared to those fed PO. Hemopexin (HPX) is a glycoprotein in plasma which binds to free heme and; therefore, plays an important role in detoxifying heme and lipid oxidation [44]. Plasma HPX level is; therefore, an important biomarker for inflammatory and oxidative stress [45,46]. Our results showed that dietary PO increased plasma HPX levels. This suggests that dietary SFAs lead to increased HPX levels in plasma, indicating that HPX is necessary to attenuate heme lipid-oxidative capacity by SFAs. These findings suggest that all lipid sources might influence coagulation pathways. Both n-3 and n-6 PUFAs appear to promote health in Nile tilapia, compared with SFAs, via elevation of coagulation proteins and lowering of heme lipid-oxidative capacities.

Proteome analysis also showed that a number of proteins related to several biological processes were modulated by different DLs. For instance, zinc finger proteins are involved in various biological processes including transcriptional regulation, DNA recognition and lipid binding. In addition, zinc finger proteins are involved in the Golgi morphology and function [47] and interaction of zinc finger protein with lipids facilitates several biological processes. For example, several zinc finger proteins such as zbtb20 and ZNF202 are associated with lipid metabolism [48,49]. Leydig cell tumour protein homolog (c19orf53) is associated with various biological pathways, such as rRNA biogenesis, ovarian cancer and replication of Zika virus [50,51,52]. Cadherins are transmembrane proteins which play an important role in cell-cell adhesion, morphogenesis and maintenance of cellular structure [53,54]. Our observations showed that dietary PO and LO significantly increased plasma ZNF501 and c19orf53. In addition, dietary SBO led to an increase in blastomere cadherin-like. Similarly, gamma-linolenic acid was reported to induce expression of E-cadherin in human cancer cells, and a reduction in E-cadherin was observed in DHA-treated cell lines [55,56]. These findings suggest that SFAs, n-6 PUFAs and n3-PUFAs are associated with various biological processes via these proteins, which warrant further investigation.

In addition to the findings related to the comparative proteomic analysis, this study demonstrated the effect of different DLs on the expression of genes in liver and muscle that were identified from the proteome. Our results showed that the expression levels of mRNAs in liver and muscle, and levels of proteins in the plasma were not correlated, with the exception of *c19orf53* and *znf501*. Indeed, dissimilar transcription of genes and protein abundances is in agreement with the findings of previous studies [57,58]. The distinct patterns of mRNA and protein levels are potentially due to the sampling time after induction [59], which results in different levels of transcripts and translated protein. In addition, the plasma protein levels might be translated from other tissues which is a presentation of whole-body protein expression, not only liver and muscle. Moreover, the half-life of mRNA in particular tissues, and variable post-translational processes might partly explain the differences between mRNA and its corresponding protein levels.

## 5. Conclusions

While different DLs had no effects on haematological parameters, all DLs increased humoral immune processes, via A2ML and/or ACH50. Our findings also raise hypotheses for further investigation that both n-6 and n-3 PUFAs appeared to promote coagulation activity via the increase of FGG, KNG1 and HRG. In addition, SFAs appear to be involved in heme lipid-oxidation (as indicated by HPX expression). Therefore, different lipid sources, which include distinct FAs have an impact on several parameters related to the health status of Nile tilapia.

## Figures and Tables

**Table 1:** Feed ingredients and chemical composition of the experimental diets containing palm oil (PO), linseed oil (LO) or soybean oil (SBO).

Ingredient (%)	Diet		
	PO	LO	SBO
Casein	32.0	32.0	32.0
Gelatin	6.0	6.0	6.0
Palm oil	10.0	-	-
Linseed oil	-	10.0	-
Soybean oil	-	-	10.0
Dextrin	30.0	30.0	30.0
Dicalcium phosphate	1.0	1.0	1.0
Premix ^1^	4.0	4.0	4.0
Carboxymethyl cellulose	2.0	2.0	2.0
Alpha-cellulose	15.0	15.0	15.0
*Proximate composition* (%)			
Dry matter	81.3	81.5	81.8
Crude protein	28.3	28.3	28.3
Crude lipid	10.7	10.4	10.6
Crude fibre	2.7	2.8	2.8
Crude Ash	3.2	3.3	3.3
Nitrogen-free extract ^2^	36.4	36.7	36.8

^1^ Vitamin and trace mineral mix provided the following (IU kg^−1^ or g kg^−1^ diet): Biotin, 0.25 g; folic acid, 0.003 g; inositol, 0.25 mg; niacin, 0.0215 g; pantothenic acid, 0.03 g; vitamin A, 5000 IU; vitamin B1, 0.0025 g; vitamin B2, 0.0012 g; vitamin B6, 0.0075 g; vitamin B12 0.00005 mg; vitamin C, 1 g; vitamin D3, 1000 IU; vitamin E, 100 IU; vitamin K, 0.008 g; copper, 0.02 g; iron, 0.2 g; selenium, 0.3 mg; zinc, 0.32 g. ^2^ Nitrogen-free extract = dry matter (crude protein + crude lipid + crude fibre + ash).

**Table 2:** Primers used for quantification of relative gene transcript expression, annealing temperature used for qRT-PCR reaction and expected amplicon sizes.

Gene Transcript Name (Abbreviation)	GenBank Accession Number	5′ to 3′ Nucleotide Sequences^1^	Annealing Temperature (°C)	Expected Amplicon Size (bp)
*CDH*	XM_005465470.2	5′-AGGCCAAAGATGATGATGAGCCAG-3′ 5′-TCCCGCCATATCTGCTGCTTGTAC-3′	58	200
*FGG*	XP_013129545.1	5′-GTTTCTACTTCGGTGGAGATGCAG-3′ 5′-GCAGCATGACATCTGTTCATCCAC-3′	55	109
*HPX*	XP_003440941.1	5′-CCTCAAGGAGAGTGCATTACCGAC-3′ 5′-GAACCTGGTGAAGTTGTGTCCATG-3′	60	198
*A2ML*	XP_005461528.1	5′-GGAAGAGGACCAGGAAACACTTGG-3′ 5′-ACACCACCCTTCATTCTGTTGTTG-3′	55	187
*KNG1*	XP_005464291.1	5′-TGCCACAGCTACATCTTCACCCTC-3′ 5′-GGCTTCAAAGCTTTCCAGGGCTTG-3′	55	162
*HRG*	XP_005475829.1	5′-GATGGTGATGCCACCATAACCA-3′ 5′-CGTCCCACTTCCTTCAAGATGTAG-3′	58	202
*ZNF501*	XM_005477455.2	5′-CTGAAGAGACACATGGCACTTCAC-3′ 5′-CCTGTGTGAACTCTGATGTGCGTC-3′	58	198
*C19orf53*	XM_003455758.4	5′-CGAAGAAAGGAGGGAGGATCATTG-3′ 5′-TTAATCACACTCAGCGGCTTGTGG-3′	55	157

^1^ The first row of the sequence denotes the forward primer, and the second row is the reverse primer.

**Table 3:** Haematological and immune parameters of Nile tilapia fed experimental diets containing palm oil (PO), linseed oil (LO) or soybean oil (SBO) for 90 days. Means with different superscripts in each column differ significantly from each other (*p* < 0.05; one-way ANOVA).

Parameters	Diet		
	PO	LO	SBO
*Haematological parameters*			
RBC^1^ (cells × 10^6^ µL^−1^)	1.7 ± 0.2	2.3 ± 0.3	2.0 ± 0.4
Haemoglobin (g dL^−1^)	8.9 ± 0.5	9.7 ± 1.3	8.8 ± 0.6
Haematocrit (%)	29.1 ± 0.5	31.6 ± 3.9	29.1 ± 1.3
*Immune parameters*			
Lysozyme activity (µg mL^−1^)	11.32 ± 1.16	12.34 ± 0.73	11.73 ± 0.60
Total immunoglobulin (mg L^−1^)	2.89 ± 0.31	2.57 ± 0.13	2.58 ± 0.34
ACH50^2^ (units mL^−1^)	94.82 ± 11.19 ^a^	128.20 ± 17.37 ^b^	105.02 ± 26.92 ^ab^

^1^ RBC = red blood cell number; ^2^ACH50 = alternative complement haemolytic 50.

**Table 4:** Differential protein spots in the plasma of Nile tilapia fed experimental diets for 90 days.

Spot No.	Protein [Species]	Entry Name (Uniprot)	Mascot Score	GI Number ^a^	Mw_t_/pI_t_^b^ (Mw_e_/pI_e_^c^)	Sequence Coverage (%)	Number of Matched Peptides	Best Peptide Match: Sequence
*Immune, inflammatory and coagulation*								
128	Alpha-2-macroglobulin-like [*O. niloticus*]	A2ML	78	gi|542250738	74,969/5.64 (73,451/4.8)	18	5	MSIFTKMEQK
288	Kininogen-1-like isoform X1 [*O. niloticus*]	KNG1	122	gi|542257849	41,185/5.80 (66,931/4.4)	44	5	AVHSAVDKFNER
821	Hemopexin-like [*O. niloticus*]	HPX	84	gi|348506792	50,278/5.91 (37,438/5.3)	4	2	IRDVDLSATPR
835	Fibrinogen gamma chain [*O. niloticus*]	FGG	83	gi|908456888	42,405/4.80 (38,318/5.2)	35	3	SSDPDVHFK
886 400	Histidine-rich glycoprotein-Like [*O. niloticus*]	HRG ^d^	117 94	gi|542204170 [gi|908518607]	48,193/6.64 (36,015/5.2) [39,519/6.52] [(62,423/6.2)]	33 31	6 4	FRLHEVQGNSVEQVDGGCNVK K.AVHHINEYHDHGYK.F
*Cytoskeleton-related proteins*								
477 459	Blastomere cadherin-like [*O. niloticus*]	CDH ^d^	120 102	gi|542261060	64,731/4.67 (55147/4.6)	35 10	7 3	KLFYSISGPGADLPPVDR [R.VFPDVFFTENNR.G]
*Miscellaneous paths*								
343	Leydig cell tumour 10 kDa protein homolog [*Ictalurus punctatus*]	C19orf53	343	gi|1042336086	10,905/11.63 (63,891/4.7)	30	4	KQLNKPK
796	Zinc finger protein 501 [*Sinocyclocheilus rhinocerous*]	ZNF501	54	gi|1025318457	44,576/8.99 (40,768/4.9)	12	4	VKTEFIK
980	uncharacterized protein KIAA2012-like isoform X1 [*Lepisosteus oculatus*]		51	gi|973180948	91,103/5.93 (33,331/5.5)	7	6	R.TLRDLTGAILAYGNK.Q

^a^ GI number = accession number in National Center for Biotechnology Information (NCBI) database. ^b^ Mw_t_/pI_t_ = theoretical molecular weight/theoretical isoelectric point. ^c^ Mw_e_/pI_e_ = experimental molecular weight/experimental isoelectric point. ^d^ Same identification was found in two spots.

**Table 5:** Hepatic and muscular mRNA levels of the corresponding proteins in plasma of Nile tilapia that were fed experimental diets for 90 days.

Target Genes	Diet		
	PO	LO	SBO
**Liver**			
*a2ml*	0.76 ± 0.05	0.73 ± 0.04	0.69 ± 0.04
*kng1*	0.72 ± 0.06 ^b^	0.64 ± 0.03 ^a^	0.60 ± 0.06 ^a^
*hpx*	0.59 ± 0.08	0.50 ± 0.01	0.53 ± 0.07
*fgg*	0.73 ± 0.04	0.71 ± 0.03	0.70 ± 0.02
*hrg*	0.22 ± 0.03 ^b^	0.16 ± 0.04 ^ab^	0.12 ± 0.06 ^a^
*cdh*	0.33 ± 0.02 ^b^	0.30 ± 0.00 ^a^	0.30 ± 0.01 ^a^
*c19orf53 ^1^*	0.41 ± 0.04	0.38 ± 0.02	0.37 ± 0.01
*znf501 ^1^*	0.60 ± 0.02 ^b^	0.56 ± 0.02 ^a^	0.56 ± 0.00 ^a^
**Muscle**			
*a2ml*	0.48 ± 0.14	0.46 ± 0.14	0.53 ± 0.05
*kng1*	0.42 ± 0.03 ^a^	0.49 ± 0.07 ^b^	0.51 ± 0.04 ^b^
*hpx*	0.20 ± 0.12 ^a^	0.27 ± 0.11 ^ab^	0.37 ± 0.04 ^b^
*fgg*	0.35 ± 0.03	0.41 ± 0.10	0.44 ± 0.04
*cdh*	0.23 ± 0.14	0.24 ± 0.08	0.28 ± 0.03
*c19orf53*	0.57 ± 0.03	0.58 ± 0.06	0.53 ± 0.02
*znf501*	0.38 ± 0.05 ^a^	0.47 ± 0.09 ^ab^	0.49 ± 0.04 ^b^

Means with different superscripts in each row differ significantly from each other (*p* < 0.05). The mRNA level of each gene was normalized to the *18s rRNA* level after log10 transformation of each level. ^1^ Note that significant correlation between protein amount and mRNA level were observed.

## Supplementary Materials

The following are available online at https://www.mdpi.com/2076-2615/10/8/1377/s1, Table S1: Predicted biological functions of the identified proteins.
