# Supplementary material for: Effects of Different Dietary Vegetable Lipid Sources on Health Status in Nile Tilapia (Oreochromis niloticus): Haematological Indices, Immune Response Parameters and Plasma Proteome
Source: Animals (Basel). 2020 Aug 8;10(8):1377. doi: 10.3390/ani10081377 (PMC7460521; doi:10.3390/ani10081377)
Supplement: Supplementary file 1 [file animals-10-01377-s001.pdf]

Table S1. Predicted biological functions of the identified proteins.

Spot number	Protein name of highest mascot score hit (Gene name, GI: accession)	Molecular function GO terms associated	Biological process GO terms associated	Cellular component GO terms associated
Immune, inflammatory and coagulation				
128	Alpha-2-macroglobulin-like	Serine-type endopeptidase inhibitor activity (GO:0004867)<br>Peptidase inhibitor activity (GO:0030414)	Liver development (GO:0001889)	Extracellular space (GO:0005615)
288	Kininogen-1-like-isoform X1	Cysteine-type endopeptidase inhibitor activity (GO:0004869)<br>Signaling receptor binding (GO:0005102)<br>Protein binding (GO:0005515)	Platelet degranulation (GO:0002576)	Extracellular region (GO:0005576)
400,886	Histidine-rich glycoprotein-like	Cysteine-type endopeptidase inhibitor activity (GO:0004869)<br>Signaling receptor binding (GO:0005102)<br>Protein binding (GO:0005515)	Angiogenesis (GO:0001525)<br>Platelet degranulation (GO:0002576)<br>Positive regulation of immune response to tumor cell (GO:0002839)<br>Chemotaxis (GO:0006935)	Extracellular region (GO:0005576)<br>Plasma membrane (GO:0005886)
821	Hemopexin-like	Protein binding (GO:0005515)	Positive regulation of immunoglobulin production (GO:0002639)<br>Positive regulation of humoral immune response mediated by circulating immunoglobulin (GO:0002925)	Extracellular region (GO:0005576)
835	Fibrinogen gamma chain	Signaling receptor binding (GO:0005102)<br>Structural molecule activity	Toll-like receptor signaling pathway (GO:0002224)	Extracellular region (GO:0005576)

		(GO:0005198)<br>Extracellular matrix structural constituent (GO:0005201)<br>Protein binding (GO:0005515)	Platelet degranulation (GO:0002576)	
Cytoskeleton-related proteins				
459,477	Blastomere cadherin-like	Calcium ion binding (GO:0005509)<br>Protein binding (GO:0005515)<br>Regulation of gene expression (GO:0010468)	Calcium ion transport (GO:0006816)<br>Homophilic cell adhesion via plasma membrane adhesion molecules (GO:0007156)	Plasma membrane (GO:0005886)
Miscellaneous paths				
343	Leydig cell tumor 10 kDa protein homolog	-	-	Nucleus (GO:0005634)
796	Zinc finger protein 501	DNA-binding transcription factor activity, RNA polymerase II-specific (GO:0000981)<br>Protein binding (GO:0005515)<br>Metal ion binding (GO:0046872)	Negative regulation of transcription by RNA polymerase II (GO:0000122)<br>Positive regulation of transcription by RNA polymerase II (GO:0045944)	Nucleoplasm (GO:0005654)
